# Strategies to solve the reverse inference fallacy in future MRI studies of schizophrenia: a review

**DOI:** 10.1007/s11682-020-00284-9

**Published:** 2020-04-17

**Authors:** Chuanjun Zhuo, Gongying Li, Xiaodong Lin, Deguo Jiang, Yong Xu, Hongjun Tian, Wenqiang Wang, Xueqin Song

**Affiliations:** 1 Department of Psychiatry, The First Affiliated Hospital of Zhengzhou University, 450000 Zhengzhou, China; 2 Department of Psychiatry Pattern Recognition, Department of Genetics Laboratory of Schizophrenia, School of Mental Health, Jining Medical University, 272119 Jining, China; 3 Department of Psychiatry, Wenzhou Seventh People’s Hospital, 325000 Wenzhou, China; 4 Department of Psychiatry, First Hospital/First Clinical Medical College of Shanxi Medical University, Taiyuan, China; 5 MDT Center for Cognitive Impairment and Sleep Disorders, First Hospital of Shanxi Medical University, 030001 Taiyuan, China; 6 Department of Psychiatric-Neuroimaging-Genetics and Co-Morbidity Laboratory (PNGC_Lab), Tianjin Anding Hospital, Tianjin Mental Health Center, Tianjin Medical University Mental Health Teaching Hospital, 300222 Tianjin, China; 7 Biological Psychiatry of Co-collaboration Laboratory of China and Canada, Xiamen Xianyue Hospital, University of Alberta, Xiamen Xianyue Hospital, 361000 Xiamen, China; 8 Department of Psychiatry, Tianjin Medical University, 300075 Tianjin, China; 9 Psychiatric-Neuroimaging-Genetics-Comorbidity Laboratory (PNGC_Lab), Tianjin Anding Hospital, Department of Psychiatry, Tianjin Mental Health Centre, Mental Health Teaching Hospital of Tianjin Medical University, Shanxi Medical University, 300222 Tianjin, China

**Keywords:** Schizophrenia, Bridges, Reverse inference, Multidisciplinary technologies

## Abstract

Few advances in schizophrenia research have been translated into clinical practice, despite 60 years of serum biomarkers studies and 50 years of genetic studies. During the last 30 years, neuroimaging studies on schizophrenia have gradually increased, partly due to the beautiful prospect that the pathophysiology of schizophrenia could be explained entirely by the Human Connectome Project (HCP). However, the fallacy of reverse inference has been a critical problem of the HCP. For this reason, there is a dire need for new strategies or research “bridges” to further schizophrenia at the biological level. To understand the importance of research “bridges,” it is vital to examine the strengths and weaknesses of the recent literature. Hence, in this review, our team has summarized the recent literature (1995–2018) about magnetic resonance imaging (MRI) of schizophrenia in terms of regional and global structural and functional alterations. We have also provided a new proposal that may supplement the HCP for studying schizophrenia. As postulated, despite the vast number of MRI studies in schizophrenia, the lack of homogeneity between the studies, along with the relatedness of schizophrenia with other neurological disorders, has hindered the study of schizophrenia. In addition, the reverse inference cannot be used to diagnose schizophrenia, further limiting the clinical impact of findings from medical imaging studies. We believe that multidisciplinary technologies may be used to develop research “bridges” to further investigate schizophrenia at the single neuron or neuron cluster levels. We have postulated about future strategies for overcoming the current limitations and establishing the research “bridges,” with an emphasis on multimodality imaging, molecular imaging, neuron cluster signals, single transmitter biomarkers, and nanotechnology. These research “bridges” may help solve the reverse inference fallacy and improve our understanding of schizophrenia for future studies.

## Introduction

Schizophrenia, originally termed as dementia praecox by Kraepelin et al. in 1877 A.D., is a complex mental disorder characterized by hallucinations, delusions, and impaired cognitive abilities (Kraepelin et al. 1953). For more than 60 years (Fischer 1953; Georgi et al. 1954; Himwich et al. 1937), researchers have attempted to develop a biological test for diagnosing schizophrenia, which could lead to improved diagnostic rates and optimized treatment strategies. However, thousands of studies have proven that the discovery of biomarkers and the creation of biological tests for neurological disorders are challenging (Kapur 2011).

For the last 50 years, the number of research studies on the genetics of schizophrenia has gradually increased, leading to the development of genome-wide association studies (GWAS) about 24 years ago (Huxley et al. 1964). However, the complexity of schizophrenia and the lack of reproducibility among the studies has limited the application of GWAS findings in schizophrenia (Lin et al. 2016). Besides, some researchers believed that the Human Connectome Project (HCP), which was initiated in 2009, would sufficiently explain the pathophysiology of schizophrenia, yet the community quickly discovered that the HCP also had limitations.

Both GWAS studies and the HCP are limited by the reverse inference fallacy, in which researchers are unable to test brain alterations assessed by genes or imaging studies in the opposite direction. In other words, patients with schizophrenia may have some specific gene or structural alteration, yet individuals with this gene or structural alteration cannot be diagnosed with schizophrenia as the gene or structural alteration may have other functions. Hence, researchers are unable to use reverse inference to diagnose patients with schizophrenia or other mental disorders.

The reverse inference fallacy also plays a role in the neuroimaging of schizophrenia, specifically magnetic resonance imaging (MRI). In 1984, Smith et al. (1984) were the first to employ MRI for studying schizophrenia in patients. Since this initial investigation, more than 11,000 additional studies have explored the application of MRI for schizophrenia (Symms et al. 2004). Two types of MRI, including structural and functional MRI (fMRI), are used in the clinic. While structural MRI is used to assess anatomical structures, fMRI is used to view the metabolic function of tissues through the detection of oxygen levels.

It is time that we, as researchers, consider schizophrenia in its entirety, rather than its individual components. This can be accomplished using multidisciplinary technologies to study the disease under the guidance of the reconstruction theory. In this review, we emphasized the need to reconstruct the overall research system of schizophrenia instead of splicing it into different disciplines, which splits the whole concept of schizophrenia. Since Insel (2010), the most famous psychiatrist of this century, suggested the need to “rethink schizophrenia,” many scholars have used genetics, epigenetics, and brain imaging techniques to study schizophrenia as a neurodevelopmental disease. Many hypotheses have been established, such as schizophrenia is a disorder of neurological connections. However, the theories have only explained a portion of schizophrenia cases, and a universally accepted hypothesis remains to be identified. More importantly, there have been no substantial improvements in the treatment of schizophrenia. Therefore, we need to rethink our current approach to the study of schizophrenia. In this review, we summarized the results of image studies of schizophrenia in the past 30 years and put forward the hypothesis that research “bridges” may advance the future study of schizophrenia.

## Literature Search

Web of Science was searched using the following search terms: MRI, structural MRI, functional MRI, schizophrenia, first-episode schizophrenia, chronic schizophrenia, and others as needed. The inclusion criteria were: (1) articles published since 1995 and (2) articles published in journals with an impact factor of 5 or above. The exclusion criteria were: (1) articles not in the English, (2) articles published before 1995, (3) articles lacking healthy controls, (4) articles missing human study approval information, and (5) articles published in journals with an impact factor below 5. An impact factor of 5 was used as the cut-off to increase the integrity of this review and ensure that articles were highly impactful and regarded by the research community.

## Structural MRI for the assessment of schizophrenia

There are a few comprehensive reviews about the structural alterations associated with schizophrenia (Lawrie and Abukmeil 1998; Shepherd et al. 2012). However, the meta-analyses have shown that it is difficult to compare the data between studies as several factors can affect the imaging results. For example, the scanner type and strength, imaging sequence, post-processing procedures, slice thickness, and measurement protocols vary between studies and insert some innate degree of variability in the studies. For these reasons, comparative analyses between studies have been complicated.

### Structural brain alterations associated with schizophrenia

The assessment of structural brain alterations provides insight into the disease etiology and pathology. Since the advent of molecular imaging, researchers have aimed to uncover specific structural alterations in many neurological disorders. The common and distinct structural alterations detected in patients with schizophrenia are shown in Table 1.

**Table 1:** List of structural brain alternations associated with schizophrenia with the respective outcomes, related conditions, and frequency.

brain Region	Alteration	Outcome	Related Conditions	Frequency	References
Whole brain	Reduced gray & white matter volume; increased CSF	Decreased cognitive performance	None	Very common	(Wright et al. 2000; Cannon et al. 1998)
Ventricles	Enlarged third and/or lateral ventricles	Communication hindrances	AD	Very common	(Steen et al. 2006; Nestor et al. 2008)
Frontal Lobe	Reduced inferior PFC volume	Impaired value processing and executive functioning	ADHD; BPD; CP; MDD;	Very common	(Walton et al. 2018; Cauda et al. 2014)
Temporal Lobe	Medial TL volume reductions of the amygdala, hippocampus and parahippocampal gyrus, superior temporal gyrus and cortex; asymmetry of the supramarginal gyrus	Impaired hearing and selective listening	ADHD; ASD	Common	(Shepherd et al. 2012; Goldstein et al. 1999; Velakoulis et al. 2006; Gaser et al. 2004)
Parietal Lobe	Reduced gray matter volume in all parietal sub-regions; decreased gray matter volumes in left parietal postcentral region; reduced white matter volumes	Decreased memory, visual and spatial processing, movement control, knowledge of numbers, sensations	ADHD; ASD	Common	(Jonides et al. 1998; Vaidya 2012; Ha et al. 2015; Kyriakopoulos et al. 2009; Zhang et al. 2017)
Occipital Lobe	Reduced gray matter volume overall	Visual impairments	GAD	Uncommon	(Xia et al. 2017; Zhang et al. 2011)
Diencephalon	Reduced volume in thalamus and hypothalamus	Problems with perception, encoding, retrieval, and prioritization of information	MDD	Common	(Connor et al. 2011; Ancelin et al. 2019)
Cerebellum	Reduced volumes of total cerebellum, left cerebellar hemisphere, and right vermis; reduced volume of striatum; increased vermis volume	Problems with gait, balance, and fine motor control	ASD; ADHD	Uncommon	(Kim et al. 2018; Vaidya 2012; Konarski et al. 2005)

AD, Alzheimer’s disease; ADHD, Attention-deficit/hyperactivity disorder; ASD, Autism spectrum disorder; BPD, Bipolar disorder; CP, Chronic pain; Generalized anxiety disorder; MDD, Major depressive disorder

The most common structural alteration is ventricular enlargement, primarily of the third and lateral ventricles. The enlarged lateral ventricular volumes are often associated with cortical atrophy. Although ventricular enlargement is observed in more than 80% of schizophrenia cases, there is some disagreement on which ventricle is most associated with disease progression (Lawrie et al. 2011). Currently, lateral ventricle enlargement is the most common findings in patients with schizophrenia, followed by third ventricle enlargement. No studies have reported abnormalities of the fourth ventricle (Jacobsen et al. 1997). In a recent 20-year follow-up study, Sayo et al. (2012) compared the ventricular volumes of patients with schizophrenia versus healthy controls in 72 studies and found that ventricular enlargement was the most prominent neurological biomarker of schizophrenia. However, the authors cautioned readers about the need for homogeneity in study protocols.

In addition to ventricular enlargement, structural alterations have also been detected in the frontal lobe. For example, reduced gray matter volumes have been detected in the left ventrolateral prefrontal cortex (PFC) and right dorsolateral PFC (Spalletta et al. 2014). In addition, gray matter loss in cortex loss in the anterior temporal lobe, along with medial frontal lobe changes and inferior frontal lobe volume. Lastly, cortical thinning in several regions, including the PFC, has been found in some patients with schizophrenia. In a recent study, Walton et al. (2018) uncovered a strong correlation between cortical thinning in the PFC and negative symptoms in patients with schizophrenia. Cortical thinning may be a potential biomarker for schizophrenia as it is not found in patients with psychotic or non-psychotic bipolar type I disorder (Godwin et al. 2018). Interestingly, some studies have shown that gray matter volumes in the frontal and temporal cortices were negatively correlated with delusional symptoms. Hallucinations have been negatively correlated with gray matter volume in the bilateral frontal and temporal cortices.

Structural alterations have also been detected in the temporal and parietal lobes of patients with schizophrenia (Poeppl et al. 2016; Tohid et al. 2015). Several studies have shown gray matter loss throughout all parts of the parietal lobe. There have also been reductions in cortical thickness of the parietal and frontal regions in schizophrenia. Many of these changes are present during the early stages of the disease and may be directly correlated with the enlargement of the ventricles. Currently, there is limited knowledge about the involvement of the parietal lobe in schizophrenia. However, it is safe to assume that the parietal lobe is altered by the disease as it is involved in many of the neurological functions affected by the disease (Jonides et al. 1998). Lastly, patients with adult-onset schizophrenia have been shown to have more extensive white matter deficits of the parietal lobe as compared with adolescent-onset schizophrenia (Kyriakopoulos et al. 2009).

Lastly, several alterations of the cerebellum have been detected in patients with schizophrenia, yet these findings have been inconsistent. While some researchers have identified cerebellar gray matter volume reductions in patients with schizophrenia (Rasser et al. 2010), others have shown volume increases in specific regions of the cerebellum, such as the vermis (Levitt et al. 1999). Moberget et al. (2018) assessed more than 1,000 patients with schizophrenia and found that cerebellar volumes were significantly reduced in patients with schizophrenia, independent of age or disease duration. These findings were verified and expanded to further pinpoint the areas of volume change, including the cerebellar lobules VI and X, in patients with first-episode schizophrenia (Kim et al. 2018). The connection between schizophrenia and occipital lobe alterations has not been fully established. However, changes in the gray matter, white matter, and occipital lobe volumes have been detected in patients with schizophrenia (Tohid et al. 2015).

Whole-brain volumes and general gray and white matter volumes have been used to differentiate patients with schizophrenia from healthy controls. Interestingly, only a few studies have quantified the changes in size. For example, Olabi et al. (2011) determined the annualized percentage volume changes in the whole brain, whole-brain gray matter, frontal white matter, and temporal white matter were − 0.07, -0.59, -0.32, and − 0.39 in patients with schizophrenia, respectively. However, the bilateral lateral ventricles showed an enlargement of + 0.36%.

### Consistency of structural brain alterations in patients with schizophrenia

Schizophrenia is a degenerative neurological condition that affects most individuals for their entire lives. Hence, the impact that disease duration may have on the structural properties of the brain must be considered. Some studies have assessed patients during the initial stage of schizophrenia, which is often called first-episode schizophrenia. However, the majority of imaging studies have been conducted in medicated patients with chronic schizophrenia, as these individuals are the most readily accessible to researchers. Patients with first-episode schizophrenia who are non-medicated hold excellent research value to the research community for three reasons. First, these patients provide a unique opportunity for researchers to investigate the disease in the absence of two significant confounding factors, including disease duration (i.e., chronicity) and drug exposure. Next, patients in the initial stages of schizophrenia can provide further insight into the possible etiology of the disease, which will aid in the development of future therapies. Lastly, these patients have structural and functional brain changes likely different from those individuals with chronic schizophrenia, which allows for the assessment of how time affects the prognosis of schizophrenia.

In comparison to healthy controls, patients with first-episode schizophrenia have enlarged third and lateral ventricular volumes, increased hippocampal volumes, and reduced whole-brain volumes (Ebdrup et al. 2010). While ventricular enlargement has been detected in most patients with first-episode schizophrenia, the connection between the third and lateral ventricle involvement remains unclear. Previously, Fannon and colleagues (2000) found enlargement of both ventricles in patients with first-episode psychosis, which was associated with developmental delays. However, Cahn et al. (2002) further examined the structural abnormalities of patients and found that enlargement of the third ventricle was the only positive characteristic of patients with first-episode schizophrenia, despite measuring the total brain, frontal lobe, hippocampus, parahippocampal gyrus, thalamus, caudate, and lateral ventricle volumes. These findings suggest that ventricular enlargement may be a fundamental structural alteration that occurs during the initial stages of schizophrenia, which is followed by other alterations during advanced stages of the disease. However, this finding has been questioned by some researchers, such as Berger et al. (2017), who recently assessed ventricular volumes across different stages of schizophrenia and found that ventricular enlargement was not a characteristic feature of early-stage schizophrenia. While nearly all studies have found ventricular enlargement to be an essential characteristic of first-episode schizophrenia, additional research is needed to assess the connection between third and lateral ventricular involvement. In addition, genetics likely play a role as siblings of patients with schizophrenia also show signs of third ventricle enlargement (Staal et al., 2000).

In a recent study, Dazzan et al. (2015) found that structural alterations in the medial temporal and PFC areas correlated with poor symptomatic and functional outcomes in patients with first-episode schizophrenia. Patients with first-episode schizophrenia were found to have gray matter reductions in the subcortical structures, including the caudate head and thalamus. The cortical gray matter reductions were located in several areas, including the insula, anterior cingulate gyrus, inferior frontal gyrus, and limbic gray matter reductions were found in the uncus and amygdala. Interestingly, gray matter volume was higher in the left putamen, a region of the brain associated with movement regulation and learning abilities. In another study, Wu et al. (2018) found more diffused gray matter reductions in the frontal and temporal lobes of medicated patients with chronic schizophrenia, as compared with unmedicated patients with first-episode schizophrenia.

In patients with chronic schizophrenia, gray matter reductions in the thalamus have been detected (Tamagaki et al. 2005). In addition, cortical gray matter reductions were found in the insula, anterior cingulate gyrus, left inferior temporal gyrus, fusiform gyrus, and right superior and middle temporal gyrus. There were limbic gray matter reductions in the left uncus and amygdala region and the right hippocampus, while gray matter increases were found in the right and left putamen. Gray matter reductions were more apparent in patients with first-episode schizophrenia in the caudate head and left uncus. Additionally, gray matter reductions were higher in the medial frontal gyrus and left dorsolateral PFC in patients with chronic schizophrenia. However, gray matter reductions were more extensive in chronic schizophrenia than first-episode schizophrenia, especially in the cortical regions, as opposed to the limbic and cerebellar regions. Despite a substantial number of studies published on schizophrenia, the heterogeneity of study designs has made it difficult to interpret these findings.

There are several excellent reviews on structural brain alterations at different stages of schizophrenia. For example, Dietsche et al. (2017) conducted a selective review of MRI studies to assess brain changes in three groups of patients, including individuals at high-risk of developing psychosis, patients with first-episode schizophrenia, and patients with chronic schizophrenia. Their findings demonstrated that as the disease progresses into chronic schizophrenia, the reductions of cortical gray matter in the superior temporal and inferior frontal regions become more extensive. In addition, patients with first-episode psychosis were found to have a significant reduction in several gray matter regions with time, including the thalamus and frontal areas, as well as progressive cortical thinning of the superior and inferior frontal cortexes. Lastly, gray matter reductions were more substantial in patients with chronic schizophrenia, primarily in the frontal and temporal areas, thalamus, and cingulate cortices, which was especially true in patients with poor outcomes.

### Structural brain network alterations associated with schizophrenia

brain regions are connected via white matter tracts, creating a highly sophisticated communication network. Network analyses provide an in-depth examination of how specific diseases may affect the connectivity of the brain. Graph theory utilizes imaging data to uncover topological properties of brain networks by establishing nodes as brain regions, along with edges and structural or functional connections between the nodes. Structural brain connectivity has been investigated using MRI and diffusion tensor imaging (DTI) with fractional anisotropy (FA) as the primary variable. However, alternative measures can also be included, such as mean diffusivity, radial diffusivity, and axial diffusivity.

Since long-distance communication across different brain regions occurs via white matter fasciculi, it is logical to expect that alterations or breaks in these fiber nerve bundles may be associated with schizophrenia. DTI was first assessed in patients with schizophrenia in 1998 by Buchsbaum and colleagues (1998). Several abnormalities have been detected in the myelin sheaths of patients with schizophrenia, which can lead to delayed neural responses between two brain regions. For example, myelin sheath plays an important role in signal transduction, and damage to the sheath can result in significant delays in neural synchronization. As an example, dysconnectivity has been found between the frontal lobe and the temporal cortex in patients with schizophrenia (Whitford et al. 2011).

The most widely examined variable in DTI studies is FA, which represents the magnitude and directionality of water in tissues and ranges from 0 to 1. FA values are reflective of microstructural properties of the white matter tracts, with lower FA values indicating possible abnormalities with the coherence of the tracts, diameter or density of the fibers, and myelination. In a recent study, Bohlken and colleagues (2016) found that global FA was significantly correlated with an increased risk of schizophrenia. In addition, several regions were associated with local reductions in network connectivity, including the frontal, striatal, and thalamic regions. Interestingly, some brain network connections may be enhanced in individuals with schizophrenia, such as the default mode network (DMN), while other areas show reduced connectivity, including the anterior cingulate, precentral gyrus, and insula (Ji et al. 2019). The areas with reduced connectivity were involved in language processing, working memory, motor functions, and mood regulation.

The dysconnectivity hypothesis suggests that schizophrenia may result from abnormal neuronal connectivity (Stephan et al. 2009). Simply, schizophrenia may arise from abnormal connections between different regions of the brain. With this in mind, researchers have questioned which nodes may play critical roles in the neurochemical circuits whose continued dysfunction leads to further anatomical changes within the circuit as time progresses. In terms of circuits, the prefrontal-thalamic-cerebellar circuit in the most widely studied in patients with schizophrenia. This circuit is anatomically connected as the PFC is connected to the anterior and dorsomedial thalamus, while the cerebellum is connected to the cerebrum via the cerebellar peduncles. In drug naïve patients with first-episode schizophrenia, Guo et al. (2015) demonstrated that structural alterations could dramatically impact the connectivity of this circuit by disrupting bilateral causal connectivity in the sensorimotor regions. In return, the prefrontal-thalamic (limbic)-cerebellar (sensorimotor) regions likely play essential roles in the etiology of schizophrenia, yet additional evidence is needed to interpret these findings.

In another study, van den Heuvel et al. (2010) detected network alterations between the frontal and temporal lobes using DTI, which were primarily attributed to the inferior and superior frontal cortex and temporal pole. In addition, significant reductions of betweenness centrality were found, indicating that patients with schizophrenia have a reduced globally integrated structural brain network and a diminished role of central hubs. In another study, patients with schizophrenia were found to have reduced centrality of the limbic structures, yet increased connectivity between frontal and parietal clusters (Skatun et al. 2016). Further insight is needed to understand the structural networks associated with the etiology and pathophysiology of schizophrenia better.

### Structural brain alterations associated with antipsychotics

The treatment of schizophrenia is designed to alleviate the symptoms of the disease, primarily the negative symptoms. Antipsychotics are highly efficacious compounds, yet more than 25% of patients receiving antipsychotics experience one or more adverse effects, such as sedation, weight gain, or movement disorders (Correll et al. 2015). In addition, there are questions about how antipsychotics may affect the brain structurally in patients with schizophrenia. Many factors contribute to structural brain changes, such as disease duration and severity, along with the age, gender, and family history of patients.

Several reports have been published on the structural effects of antipsychotics on the brain, including an excellent review by Moncrieff and Leo in 2010 (Moncrieff and Leo 2010). Initially, first-generation antipsychotics were associated with an expansion of the basal ganglia in patients receiving treatment for first-episode schizophrenia (Scherk and Falkai 2006). However, this effect could be reversed if the patients switched to a second-generation antipsychotic. In another study, Fusar-Poli et al. (2013) found that longitudinal reductions in gray matter volume were directly associated with the use of antipsychotics. Interestingly, the brain alterations caused by antipsychotics were not associated with poor treatment outcomes and occurred even at the lowest therapeutic dosages (Emsley et al. 2017).

In a recent study, Leung et al. (2011) found that frontal, temporal, and stratio-limbic structural changes occurred during the early stage of schizophrenia and were not related to the antipsychotic treatment or illness duration. However, antipsychotic medications have been shown to contribute to structural brain changes (Scherk and Falkai 2006). Previously, Navari and Dazzan (2009) found that typical antipsychotics acted regionally on the brain, with the volumes of the basal ganglia being significantly higher in patients with schizophrenia when compared with healthy controls. This increase in basal ganglia volume has been shown in other studies and switching from typical to atypical antipsychotics was found to normalize the size of the basal ganglia (Scherk and Falkai 2006).

## Functional MRI (fMRI) for the assessment of schizophrenia

The imaging of functional activities in the brain provides additional insight into the etiology and pathology of diseases. Blood oxygenation level-dependent (BOLD) fMRI works by exerting a magnetic effect on deoxyhemoglobin, which allows for the regional and global mapping of brain regions activated by specific tasks (Chow et al. 2017). Hence, functional connectivity is estimated as the temporal associations of low-frequency oscillations of 0.01–0.10 Hz in the BOLD signal between different brain regions. A decade of studies has shown that certain conditions, such as resting-state and task-driven, produce prototypical patterns of activation in the brain. This allows for the assessment of many neural networks, including the DMN, motor network, and others in real-time. The data gained from resting-state and task-driven fMRI are useful for different purposes.

In comparison to traditional MRI, fMRI has a superior temporal resolution, spatial resolution, and reproducibility (Liu et al. 2015). Despite fMRI being a popular tool for assessing neurological processes, there have been some issues with validating the statistical methods used in fMRI in “real” datasets instead of stimulations (Eklund et al. 2016). The central problem that researchers have come across with fMRI is that abnormalities detected by fMRI are representative of general disorder conditions. While fMRI may assist physicians in determining the emergence and severity of symptoms, it does not allow for the delineation of neurological disorders from each other. For example, some of the structural and functional alterations found in patients with schizophrenia are also detected in patients with autism spectral disorder, Alzheimer’s disease, bipolar disorder, major depressive disorder, anxiety disorder, and obsessive-compulsive disorder (Sprooten et al. 2017).

### Functional brain alterations associated with schizophrenia

Functional brain alterations are those regions of the brain where the activity is higher or lower than levels found in healthy controls. The common and distinct functional brain alterations associated with schizophrenia are shown in Table 2. As shown, functional alterations have been associated with most regions of the brain to some degree, yet there is some disagreement between studies about the core functional features of schizophrenia.

**Table 2:** List of functional brain alternations associated with schizophrenia, including the outcomes, related conditions, and prevalence.

brain Region	Alteration	Outcome	Related Conditions	Frequency	References
Whole brain	Decreased overall connectivity	unknown	MDD	Uncommon	(Veer et al. 2010; Yang et al. 2014)
DMN	Spatial differences in the frontal, anterior cingulate, and parahippocampal gyri	Increased severity of symptoms	MDD; GAD	Common	(Gao et al. 2017; Zhou et al. 2016; Yan et al. 2019)
Frontal Lobe	Dysfunction of the PFC	Cognitive control deficits; memory problems	AD; ASD; BPD; MDD	Very common	(Guo et al. 2019; Ragland et al. 2009; Ragland et al. 2004, 2005; Pascual-Belda et al. 2018; Munoz-Moreno et al. 2018)
Temporal Lobe	Increased parahippocampal activation	Working memory problems	AD; ASD	Uncommon	(Guo et al. 2019; Ragland et al. 2004; Pascual-Belda et al. 2018)
Parietal Lobe	Postcentral, anterior and posterior cingulate gyri, along with precuneus and middle frontal cortex; parietal-frontal wave deficits	Decline of visual and spatial interactions	AD; MDD; PD	Common	(Alexander-Bloch et al. 2014; Zhang et al. 2017; Whalley et al. 2004; Yang et al. 2016; Geroldi et al. 2000; Mi et al. 2017; Gao et al. 2011; Bonni et al. 2013)
Occipital Lobe	Increased activation of the lingual and fusiform gyri, cuneus and precuneus	Visual processing dysfunction	unknown	Uncommon	(Ragland et al. 2005; Sehatpour et al. 2010)
Diencephalon	Reduced volume in thalamus and hypothalamus	Problems with perception, encoding, retrieval, and prioritization of information	AD; MDD; PD	Common	(Chen et al. 2019; Woodward et al. 2012; Takahashi et al. 2004; Kang et al. 2018; Dayan et al. 2018; Liu et al. 2018)
Cerebellum	Hypoactivation of the cerebellum; increased activation of striatum; altered activity of the medial frontal and anterior cingulate gyri	Cognitive, emotional, and executive processes	AD; MDD; PD	Common	(Lungu et al. 2013; Camchong et al. 2011; Gao et al. 2017; Sorg et al. 2013; Ramos et al. 2018; Simioni et al. 2016)

AD, Alzheimer’s disease; ADHD, Attention-deficit/hyperactivity disorder; ASD, Autism spectrum disorder; BPD, Bipolar disorder; CP, Chronic pain; Generalized anxiety disorder; MDD, Major depressive disorder; Parkinson’s disease

While some abnormalities have been detected in the functioning of the cerebellum, there has been little interpretation of the findings. Previously, Longo et al. (2013) reviewed 234 studies using fMRI in schizophrenia and found that 41% of the article assessed the activity of the cerebellum. Increased activation of the cerebellum was detected in approximately 66% of these studies, suggesting that it may be a common characteristic of patients with schizophrenia. However, a third of the studies did not find increased activation of the cerebellum, suggesting that the cerebellum may be an unreliable biomarker in schizophrenia.

Auditory-verbal hallucinations have been associated with gray matter reductions in the superior temporal gyrus (Lennox et al. 1999). In addition, altered functional activity has been detected in the medial frontal and arterial cingulate gyri of patients with schizophrenia (Camchong et al. 2011). Alterations in frontal connectivity were positively correlated with the severity of symptoms and cognitive abilities in patients with schizophrenia. In 2009, Minzenberg et al. (2009) analyzed changes in the executive functioning of patients with schizophrenia through a meta-analysis of 41 fMRI studies. Patients with schizophrenia displayed reduced activity in the dorsolateral PFC, anterior cingulate cortex, and thalamus. While activity increased in other areas of the PFC, these alterations were suggested to be compensatory in nature. During task performance, patients with schizophrenia had higher BOLD signals in the occipital and lateral PFC, and treatment with second-generation antipsychotics was effective in returning their working memory task performance to normal levels (Ettinger et al. 2011).

Dysfunction of the dorsolateral PFC has been linked to working memory disturbances in patients with schizophrenia, while alterations of the limbic system have been associated with facial emotion processing deficits (Manoach et al. 2000; Perlstein et al. 2001). In another study, Guo et al. (2019) found several impaired regions of activation in the brains of patients with schizophrenia, including the frontal-medial temporal lobe circuits and dorsolateral PFC, which were associated with memory deficits and cognitive problems. While several function alterations have been noted in patients with schizophrenia, many of these have also been found in patients with depression, such as increased activity in the thalamus, anterior cingulate cortex, and ventrolateral PFC (Miller et al. 2015). Lastly, a meta-analysis conducted by Gao et al. (2017) showed that anatomic and functional abnormalities of the brain were overlapped in the DMN and auditory networks in patients with schizophrenia who were drug-free at the time of the study.

### Consistency of functional brain alterations associated with schizophrenia

While there are several antipsychotics available for the treatment of schizophrenia, all of the drugs similarly target the dopamine D2 receptor. There is limited information about how altered functions progress in the brain of patients with schizophrenia. Previously, Guerrero-Pedraza et al. (2012) found that first-episode psychosis was associated with a failure of deactivation in the medial frontal cortex and was not associated with hyper- or hypo-frontality, further demonstrating the unique role of the DMN in schizophrenia.

Patients with first-episode schizophrenia also have prefrontal dysfunctions associated with context processing and disorganization (MacDonald et al. 2005). Recently, Li et al. (2017) found distinct patterns of functional dysconnectivity between patients with first-episode and chronic schizophrenia. In patients with first-episode schizophrenia, 90% of the alterations were associated with the frontal lobe, primarily the frontal gyrus. However, the thalamic alterations were significantly more prominent and global in patients with chronic schizophrenia, as reduced activity was detected in the thalamo-frontal network, along with increased thalamo-temporal and thalamo-sensorimotor connectivity. Lastly, connectivity alterations of the DMN have been primarily detected in patients with first-episode schizophrenia, yet it is believed that the DMN plays a role in disease progression (Bastos-Leite et al. 2015). Additional research is needed to determine how the disease stage and illness duration affect the functional brain alterations in patients with schizophrenia.

### Functional network alterations associated with schizophrenia

The complex network of connections between different regions of the brain allows for humans to function, yet decreased connectivity has been associated with many neurological diseases, including schizophrenia. For example, men with schizophrenia have a four-fold higher likelihood of committing violent crimes in their lifetime (Fazel et al. 2009), and this predisposition to violence may be visualized using imaging studies. As such, Hoptman and colleagues (2010) found that the functional connectivity between the amygdala and PFC was compromised significantly in patients with schizophrenia. In addition, disconnection between the frontal lobe and the amygdala has been found in several studies, as described in the systemic review by Fjellvand and colleagues (2018). As these regions play roles in aggression and social cognitive dysfunction, these alterations may be attributed to the high crime rates found in the schizophrenic population.

In addition to an increased predisposition to violence, many patients with schizophrenia experience auditory-verbal hallucinations, which are complex experiences of hearing voices in the absence of a speaker. Previously, Lawrie et al. (2002) found a disconnection between the frontal and temporal lobes connectivity in patients with schizophrenia, which was positively associated with the severity of auditory-verbal hallucinations. Interestingly, auditory-verbal hallucinations have also been associated with decreased connectivity between the temporal and parietal junction, which can also impact other aspects of the brain, including attention control, speech perception, and self-referent processing (Vercammen et al. 2010). The connectivity alterations associated with auditory-verbal hallucinations may progress as the disease progresses, as patients with the early-stage disease were found to have dysconnectivity between the frontal and occipital regions that positively correlated with the severity of auditory-verbal hallucinations (Szeszko et al. 2008). Additional research is needed to understand the brain network alterations associated with auditory-verbal hallucinations in patients with schizophrenia better.

Previous studies in schizophrenia revealed abnormalities in the cortico-cerebellar-thalamo-cortical circuit pathway, suggesting the necessity for defining thalamic subdivisions in understanding alterations of brain connectivity. Recently, Gong et al. (2019) demonstrated the loss of connectivity between several thalamic subdivisions (i.e., superior-anterior, ventromedial and dorsolateral part of the thalamus) and the sensorimotor system, anterior cingulate cortex, and cerebellum in patients with schizophrenia. A gradually increased pattern of dysconnectivity was also observed across the thalamic subdivisions, which was negatively correlated with symptom scores and duration of illness in individuals with schizophrenia.

In a study by Bitsch and colleagues (2019), patients with schizophrenia showed lower functional connectivity between the right temporo-parietal junction and temporal lobe regions, such as the hippocampus, the fusiform gyrus, and the middle temporal gyrus, indicating that the right temporo-parietal junction failed to integrate memory-informed processing during mental inferences. However, decreased functional connectivity in the right temporo-parietal junction has been detected in other neurological disorders (Poeppl et al. 2016), which makes it an unreliable marker for schizophrenia. In another study, Thompson et al. (2001) showed that patients with adult-onset schizophrenia experience parietal-frontal wave deficits prior to the onset of schizophrenic symptoms, suggesting that it may be possible to assess using MRI in high-risk patients. It was previously suggested that the parietal lobe may be the location where functional and structural alterations originate in a number of patients who develop schizophrenia, before progressing to the frontal regions during later stages of the disease (Yildiz et al. 2011). However, more research is needed to determine if this holds true in larger patient populations.

One of the most studied networks of schizophrenia is the DMN, which is an interconnected group of brain structures that remain active during resting-state MRI (Raichle 2015). Alterations in the typical DMN profile have been associated with several neurological disorders, including depression, anxiety, and schizophrenia. In patients with schizophrenia, alterations in the DMN, primarily in the frontal, anterior cingulate, and parahippocampal gyrus, have been associated with the severity of symptoms (Garrity et al. 2007). Increased DMN activity has also been detected in first-degree relatives of patients with schizophrenia and was associated with an increased risk of schizophrenia and thought disturbances (Whitfield-Gabrieli et al. 2009). Despite these findings, alterations in the DMN connectivity are non-specific and may be associated with other neurological disorders or brain injury.

### Functional brain alterations associated with antipsychotics

Neuroimaging has been used to assess how antipsychotics affect the brain as it was originally thought that antipsychotics would correct abnormal brain activity. This has been proven right in several cases. For example, treatment with antipsychotics was found to normalize the activity of lateral prefrontal regions in patients with schizophrenia to normal levels found in healthy adults (Spence et al. 1998). Recently, Cadena et al. (2018) showed that treatment with antipsychotics could improve the functional activity of the anterior cingulate cortex and ventral putamen. In addition, the authors found that early treatment was strongly connected with better treatment outcomes as patients with higher baseline functional activity of these regions showed better outcomes after treatment with risperidone.

There is some debate on the potential brain alterations induced by antipsychotics and whether long-term use of these drugs may be harmful to patients. In one study, Seitz et al. (2005) assessed the short-term effects of atypical antipsychotics on the functioning of the dorsolateral PFC and anterior cingulate cortex. Dysfunctions of the dorsolateral PFC and anterior cingulate cortex were detected in patients with first-episode schizophrenia, yet short-term antipsychotic therapy of four weeks was found to improve the functioning of the anterior cingulate cortex, demonstrating that antipsychotics can positively affect the cognitive deficits associated with schizophrenia.

In another study, Stephan et al. (2001) assessed the effects of olanzapine, a second-generation atypical antipsychotic, on the functional connectivity of the cerebellum in patients with schizophrenia. When the patients conducted a simple motor task during fMRI, the use of olanzapine resulted in significant alterations of the cerebellar functional connectivity, which was primarily located in the thalamus and PFC. Interestingly, there was minimal impact on the motor-associated structures of the brain. In another study, Sara et al. (2015) suggested that improved functional connectivity of the striatum with the limbic or prefrontal regions may be indicative of successful treatment in patients receiving antipsychotics. However, additional studies are needed to determine if fMRI can be useful for treatment monitoring. As of now, there is inadequate data to suggest that specific functional alterations can be used to assess patients before and after treatment to determine the efficacy of therapies.

## Current critical issues and future research directions

Schizophrenia is a debilitating condition that has gained significant attention around the world due to its unique set of symptoms that have been popularized by mainstream television and movies. While treatment options are available, most cases of schizophrenia go undetected for years. As the disease is progressive, the early innervation is critical for the successful treatment of patients. However, diagnosing schizophrenia is a complex process that continues to elude neurologists, neuroradiologists, and psychiatrists globally. Recently, Nemoto et al. (2020) showed that structural MRI might be useful for differentiating patients with schizophrenia from healthy controls using data from multiple institutes. However, the accuracy was low at 73%, indicating the need for further verification. It has been questioned if we are on the wrong track in discovering diagnostic tools for schizophrenia, or if we are on the right track of a highly complex problem (Maj 2011).

In a previous report, Lawrie et al. (2011) argued that, despite thousands of studies, we have little substantial evidence of the clinical utility for the pathophysiology of schizophrenia. While significant advances have been made in the field, there has been little translation of these advances into the clinic. In response to the view of Lawrie et al., Prof. Falkai (2011) suggested that the complexity of the observed phenotypes and the assessment of patients as a whole, instead of focusing on different phases of the illness, has limited the usefulness of findings from schizophrenia studies. Simultaneously, Prof. Cannon (2011) also responded to the views and suggested that researchers may be looking at the problem incorrectly. He insisted that we are unlikely to discover a simple heuristic or single diagnostic biomarker for schizophrenia, given the complexity of mental disorders and their multifaceted etiologies. Based on these opinions and others, Prof. Maj (2011) recommended that researchers rethink about how schizophrenia can be better assessed at the biological level as the biological level currently being assessed (i.e., neural circuits and neurotransmitters) is significantly different from the biological level at which answers are likely to be found.

### Current critical issue of limitations in MRI of schizophrenia

**Fig. 1:**
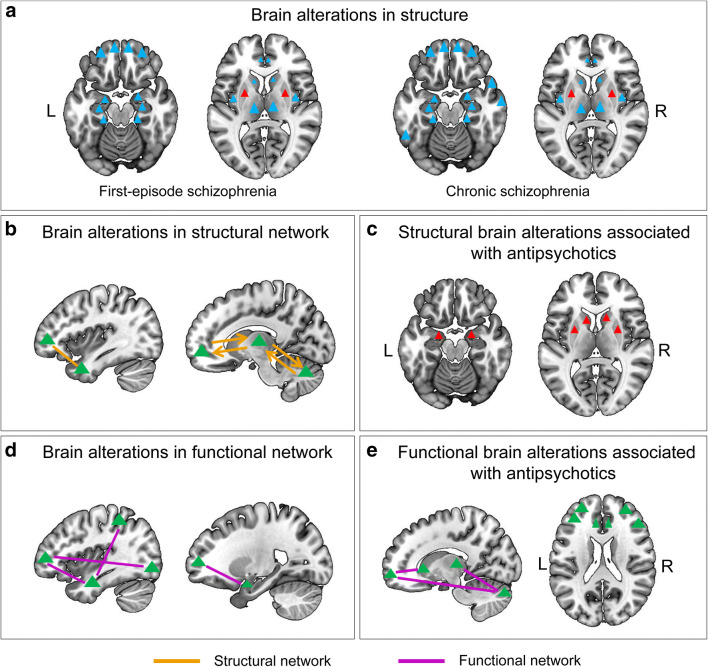
brain maps showing the common structural and functional brain alterations and the impact of antipsychotics in schizophrenia. The blue triangles represent decreased gray matter; the red triangles represent increased gray matter; the green triangles represent brain regions with abnormal structural connection, functional connection or functional alterations caused by antipsychotics; the thick green line represents the structural connection; the thick green arrows represent the circuit of structural connections; the thin green lines represent functional connections.

brain maps showing the common structural and functional alterations associated with schizophrenia, along with the effects of antipsychotics, may be found in Fig. 1. Some researchers believe that MRI studies of schizophrenia have reached their limits with our current technologies. Many of the MRI studies in schizophrenia are out of date, and there are dismal prospects in the field until new image processing methods become readily available to the research community. However, only a few research groups are working on new image processing methods at this time.

**Fig. 2:**
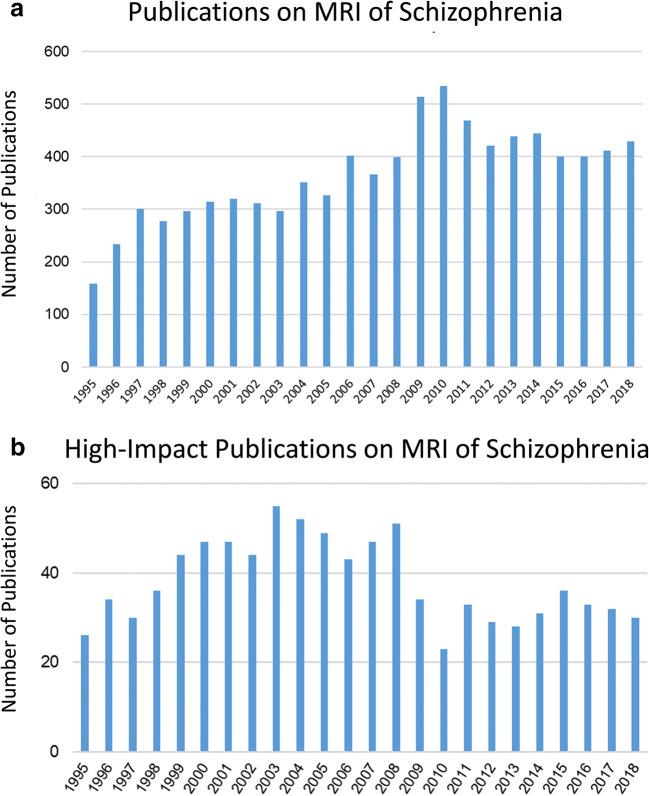
History of literature on MRI in schizophrenia. **a** Number of publications on MRI in schizophrenia has plateaued since 2010. **b** Number of articles published in high-impact journals (IF_5y_ > 5) shows that the development of MRI in schizophrenia has slowed down since 2008.

By searching the literature for MRI studies in schizophrenia, we found that the number of studies on MRI in schizophrenia plateaued in 2010 and has remained relatively stable since that time (Fig. 2a). After extracting the articles from high-impact journals (Five-Year Impact Factor, IF_5Y_ ≥ 5) from the total published literature, we found that the number of high-impact articles in the field has decreased since 2008 (Fig. 2b), further supporting our hypothesis that the development rate of this research field has slowed down. Simply, there is limited highly impactful research being published on MRI of schizophrenia at this time, likely due to technological limitations. However, some of the current limitations associated with MRI for schizophrenia are actively being addressed in the community.

Currently, there are no brain or matter-specific structures that are universally altered in patients with schizophrenia, despite gray matter abnormalities being ubiquitous in the disease. The central problem in using medical imaging to diagnose schizophrenia is the artificiality of the data available. The studies conducted up to now have primarily focused on differentiating between schizophrenia patients and healthy controls, as this is the simplest way for researchers to display their findings to the academic community. However, this strategy has an inherent flaw because it does not allow for differentiating between mental disorders with similar etiologies or symptoms. As previously mentioned by Prof. Kapur (2011), the actual dilemma is distinguishing between two patients with similar neurological profiles, such as nearly psychotic depressed-looking patients and nearly depressed psychotic-behaving individuals and being able to accurately diagnose them as schizophrenia or MDD. In addition, there are some problems with our current technologies and protocols that must be addressed. For example, Eklund et al. (2016) recently discovered fMRI false-positive rates upwards of 70% for the fMRI analysis software packages commonly used in the clinic, indicating that the interpretation of some weakly significant imaging studies may need to be reassessed.

### Current critical issue of schizophrenia and the reverse inference fallacy

**Fig. 3:**
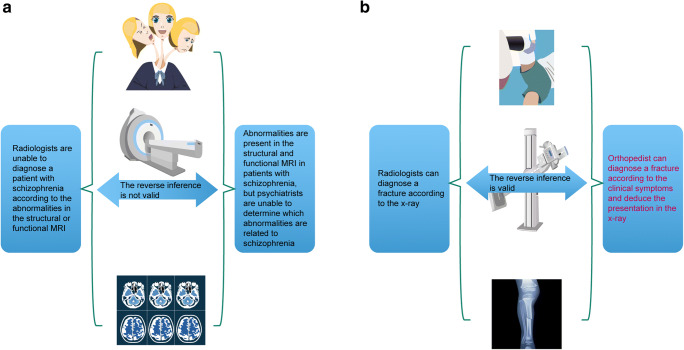
Reverse inference and its role in psychiatry. **a** Radiologists are unable to diagnose a patient with schizophrenia based on MRI, and abnormalities detected by MRI cannot be used by psychiatrists to diagnose schizophrenia. Hence, the reverse inference is not valid. **b** Radiologists can diagnose a fracture by x-ray, and orthopedists can diagnose a fracture according to clinical symptoms and deduce the presentation in the x-ray. Hence, the reverse inference is valid.

In typical neuroscience experiments, forward and reverse inference play key roles in understanding the diagnostic capabilities of MRI (Fig. 3). The forward inference is used to label a pattern from an independent variable, while reverse inference is used to label an independent variable from an activity pattern. As an example, consider a person with schizophrenia who undergoes MRI and shows specific activity patterns when asked to perform a given task, such as reciting words from their memory. Next, imagine a person with a traumatic brain injury undergoes the same MRI test and shows the same activity pattern. We cannot assume that the second patient has schizophrenia simply because the patient showed the same activity pattern as the patient with schizophrenia, as other cognitive processes caused by the brain injury could have resulted in the specific brain pattern. Hence, the reverse inference is invalid as activity patterns obtained from fMRI cannot be used to diagnose a patient with a specific neurological disorder. Simply, an activated region of the brain may be caused by a wide variety of cognitive processes, which significantly hinders the predictive powers of reverse inference. For this reason, it is important that the research community develop “bridges” that can move the research field forward into the future.

### Developing “bridges” to solve the reverse inference fallacy

**Fig. 4:**
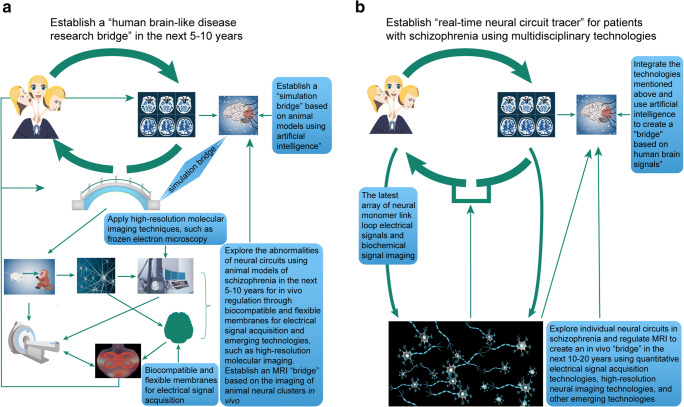
Building “bridges” to solve the reverse inference problem and advance the study of MRI in schizophrenia. **a** Establish a “human brain-like research bridge” in the next 5–10 years through artificial intelligence and new molecular imaging technologies to explore the abnormalities of neural circuits using animal models of schizophrenia. **b** Establish “real-time neural circuit tracer bridges” (Note: it is not a “real neural circuit bridge” but is only named as “maximum possible proximity to the naked truth of pathophysiology bridges”) for patients with schizophrenia using multidisciplinary technologies to explore individual neural circuits in schizophrenia.

The research field has several directions it can take to move into the future. As illustrated in Fig. 4, there is an urgent need to develop “bridges” that can eliminate the reverse inference fallacy that continues to plague the field. Some researchers have focused on the advent of correlations between the structural and functional alterations. In this type of situation, a patient could be given a diagnosis of schizophrenia if the patient meets the requirements of having a specific number of structural and functional changes (i.e., two or more structural changes in combination with three or more functional changes). For example, Chin et al. (2018) recently explored machine-learning approaches for their potential diagnostic and prognostic abilities. Specifically, they used an anatomically and spatially-regularized support vector machine framework to differentiate between schizophrenia patients and healthy individuals using whole-brain gray matter densities estimated using MRI scans. The model has an accuracy of 86.6% in the training set of 127 individuals and validation accuracy of 83.5% in an independent set of 85 individuals. By employing a sequential region-of-interest selection process, the recognition accuracy was increased to 92.0% in the training set and 89.4% in the validation set with the combination model reaching 96.6% for sensitivity and 74.1% for specificity.

However, other researchers are exploring other imaging modalities beyond or in combination with MRI. The most common dual-imaging modality is PET-MRI. In a recent study, Rao et al. (2018) utilized a radiotracer termed ^11^C-FLB 457, which was previously developed for imaging extrastriatal D2 dopamine receptors, in drug-free patients with schizophrenia. The patients were asked to complete a cognitive task during imaging, during which the researchers were able to image reductions in dopamine release within the anterior cingulate cortex and dorsolateral PFC. The combination of PET and fMRI allowed for better assessment of whole-brain global activity using novel biomarkers to assess biological processes, such as glucose metabolism (Thompson et al. 2016). Also, many advanced techniques are being used in animal studies, yet have not been translated to human studies at this time. The adoption of specific animal models with newer technologies, such as micro-optical sectioning tomography, which is capable of mapping neural circuits at the mesoscale level, and super-resolution methods like stochastic optical reconstruction microscopy (STORM) and photoactivation localization microscopy (PALM), which can image structures of 20–50 nm, may advance the study of schizophrenia. In addition, other strategies to measure electrical signals, such as nano-conjugated fluorescent probes and voltage-sensitive molecules, may be useful when combined with MRI. The combination of MRI with these other technologies can provide excellent opportunities to investigate the relationship between MRI features and neuronal cluster imaging features, which will help to understand the pathological features of schizophrenia better.

MRI may also be combined with other targeted approaches, such as nanoparticle-based electrochemical biosensors or targeted up-conversion nanoparticles, to examine neuronal cluster activity features (Blanco-Andujar et al. 2016). To improve the resolution of these findings, high-resolution 7T MRI may be used in animal and human studies. Alternatively, this could enable cellular MRI, which may provide additional insight into the etiology of schizophrenia and may be useful as a patient-specific indicator of disease progression (Makela et al. 2016). Several biomarkers have been uncovered in schizophrenia, which may play a role in future imaging studies (Lai et al. 2016). When considered together, these combination technologies may be used to transplant the “bridges” to patients to elevate the level of schizophrenia research. The combination of MRI and other imaging technologies may be useful for monitoring the effects of antipsychotics on the structure and function of the brain to optimize the current treatment strategies for schizophrenia. Lastly, the combination of GWAS and imaging studies may allow researchers to assess schizophrenia at the biological level.

Future advances in MRI research for schizophrenia will likely require interdisciplinary technologies, such as neural signal, optical imaging, and the combination of trans-synaptic tracing and optogenetics. When considering the combination of multiple technologies, such as oxygenation with fMRI, neurochemistry with nuclear magnetic resonance spectroscopy, electrodynamics with EEG, and genetic profiles by microarray analyses, for the assessment of brain physiology, we can see that the development of DTI as a white matter imaging tool provides us with an unprecedented opportunity to understand the root causes of schizophrenia, which is an understanding necessary for the targeted development of successful treatment programs. The promise of new, knowledge-based breakthroughs in the treatment of this devastating disorder makes this an exciting time to be involved in schizophrenia research.

Although most scholars have advocated for the use of advanced interdisciplinary technologies to explore the pathophysiology of schizophrenia, we must remember a famous quote. “Simply one stroke in the plan, one thread in the fabric, and the plan was called the intellectual activity and the fabric was called the education industry and neither the whole nor any of the separate specialties had the slightest value whatever (Hesse 1971).” The continued progress and radical re-visioning of the field is essential to ensure that new scientific revolutions do not become solely applications of new terminology to existing questions or socio-rhetorical phenomena rather than actual advances in science (O’Donohue et al. 2003). Inspired by these thoughts and the need to “rethink schizophrenia” by Insel, we agree that with Silverstein that radical revisioning of schizophrenia may be necessary to determine how advanced interdisciplinary technologies can be used to explore schizophrenia in new directions. It was previously recommended that researchers begin to study schizophrenia as a neurodevelopmental disease, which will likely lead to new discoveries. Accompanied by advanced interdisciplinary technologies, and inspired by the ideas of Silverstein and Insel, we propose that research “bridges” may be used to advance the study of schizophrenia. Although this is only our perspective, we believe this suggestion may aid in future studies for schizophrenia.
